# SHORT-TERM EFFECT OF PRISM ADAPTATION TREATMENT ON SEVERITY OF UNILATERAL SPATIAL NEGLECT FOLLOWING RIGHT HEMISPHERIC STROKE: A SYSTEMATIC REVIEW AND META-ANALYSIS

**DOI:** 10.2340/jrm.v57.42542

**Published:** 2025-03-24

**Authors:** Yumene NAITO, Yuta KOSHINO, Hisaaki OTA, Marianne PIANO, Akihiro WATANABE, Yuji INAGAKI, Yukina TOKIKUNI, Daisuke SAWAMURA

**Affiliations:** 1 Department of Rehabilitation Science, Faculty of Health Sciences, Hokkaido University, Sapporo, Japan; 2 Department of Occupational Therapy, School of Health Sciences, Sapporo Medical University, Sapporo, Japan; 3 Department of Optometry and Vision Sciences, Faculty of Medicine, Dentistry and Health Sciences, University of Melbourne, Melbourne, Australia; 4 National Vision Research Institute, Australian College of Optometry, Melbourne, Australia; 5 Graduate School of Health Sciences, Hokkaido University, Sapporo, Japan; 6 Department of Rehabilitation, Sapporo Shuyukai Hospital, Sapporo, Japan; 7 Department of Physiotherapy, Faculty of Medicine, Dentistry and Health Sciences, University of Melbourne, Melbourne, Australia

**Keywords:** prism adaptation, unilateral spatial neglect, stroke, meta-analysis, prism angle

## Abstract

**Objective:**

This work aimed to investigate the effects of prism adaptation on unilateral spatial neglect following right hemispheric stroke.

**Design:**

Systematic review and meta-analysis of randomized controlled trials (RCTs).

**Patients:**

Patients with unilateral spatial neglect following right hemispheric stroke.

**Methods:**

RCTs comparing prism adaptation with placebo therapy were systematically searched across 4 databases (PubMed, Web of Science, CINAHL, and Cochrane Library). Screening, data extraction, and quality assessment were performed by 2 independent reviewers.

**Results:**

A total of 7 RCTs, involving 227 participants, satisfied the eligibility criteria. The results showed significant short-term effects of prism adaptation on neglect outcomes (SMD: 0.49 [95% CI: 0.07 to 0.92], *p* = 0.02) but not on the Catherine Bergego Scale (CBS) (SMD: –0.38 [95% CI: –1.27 to 0.51], *p* = 0.40). Subgroup analyses revealed that larger prism angles (exceeding 10°) had greater prism adaptation effects on both neglect outcomes and CBS (SMD: 0.71 [95% CI: 0.30 to 1.12], *p* = 0.0007 and SMD: –0.77 [95% CI: –1.51 to –0.02], *p* = 0.04, respectively).

**Conclusion:**

This study demonstrated that larger prism angle with greater than 10° was identified as a crucial factor in eliciting prism adaptation effects. These findings support the use of prism adaptation with angles exceeding 10° as a therapeutic approach for unilateral spatial neglect.

## LAY ABSTRACT

The effectiveness of prism adaptation remains a topic of ongoing debate, despite decades of research. This systematic review and meta-analysis aimed to investigate the effects of prism adaptation on unilateral spatial neglect, a cognitive deficit where patients fail to notice or respond to contralesional stimuli. We reviewed 7 studies with 227 participants that satisfied the eligibility criteria. Our findings showed significant short-term effects of prism adaptation, especially when using larger prism angles (exceeding 10°). These results suggest that using a larger prism angle with greater than 10° could be a promising approach for unilateral spatial neglect.

Unilateral spatial neglect (USN) is a common cognitive deficit following hemispheric stroke, where patients fail to notice, respond to, or orient to contralesional stimuli. The reported frequency is higher in cases of right hemispheric stroke, with USN occurring in 70% of acute right hemispheric strokes, vs 49% of left hemispheric strokes (1). Previous studies have reported that USN is a significant risk factor for slower rate of improvement in activities of daily living (ADL) and functional independence measures, compared with people without USN (2–4). In addition, USN interferes with the recovery of physical and other cognitive functions, such as memory and executive functions (2), and is associated with longer periods of hospitalization (4) and worsening quality of life (5). Therefore, effective therapeutic approaches to ameliorate neglect symptoms are needed to improve outcomes in stroke survivors experiencing USN.

Various therapies, e.g., visual scanning training (6), mirror therapy (7), virtual reality activities (8), have been developed to try and address USN as part of stroke rehabilitation, but among these modalities, prism adaptation (PA), first reported by Rossetti et al. (9), is considered a promising approach (10, 11). During PA, patients with USN are instructed to point at a visual target while wearing ipsilesional prisms. Patients initially reach out incorrectly, but compensate for this reaching error by repeatedly recalibrating the pointing movements until accuracy is achieved (adaptation) (9). PA is a simple and non-invasive technique, and its treatment effects have been identified not only in neuropsychological tests (9, 12–14), but also in assessments related to ADL such as wheelchair navigation, walking, reading, and writing (15–18).

Existing meta-analyses on PA effects (19–23) have reported inconsistent results. Two studies have indicated that PA is effective in ameliorating USN symptoms (19, 21), while others suggested insufficient effects of PA (20, 22, 23). Furthermore, the 2 systematic reviews that did not conduct a meta-analysis (24, 25) used different outcome measures, making it challenging to discuss the consistency of PA effects. Alongside included studies that have small sample sizes, these inconsistencies may arise from variations in methodological approaches between papers: the inclusion of both left and right hemispheric stroke (22), pooling of outcome measure subscales (21), and control group assignment not being rigorous (23) (for example, allowing training other than pointing under neutral glasses). However, in general, there is insufficient evidence for consistent, beneficial effects of PA on USN and associated outcomes after stroke.

Alongside the above methodological considerations, it is important to consider some of the clinical and practical aspects that may impact PA effects. Clinically, previous studies have shown effects of PA are influenced by prism angle, disease phase, type of PA, and lesion location (18, 26–28). From a practical perspective, according to Facchin et al. (26), the 20Δ prism was found to be most effective in inducing immediate after-effects (considering 1Δ moves the field of view by 0.57°), whereas the after-effects of the 5Δ prism appeared prolonged. Ten Brink et al. (27) reported that intervention timing was also an important factor. For example, it has been suggested that it is not appropriate to evaluate PA effectiveness immediately after the onset of stroke, when symptoms fluctuate widely and are unclear (27).

It is also important to consider PA treatment protocol. PA is classified into 2 different treatment protocols, depending on hand visibility during pointing movements. In terminal PA (TPA), the patient has vision of their hand only at the final part of the pointing movement, while in concurrent PA (CPA), the patient has vision of their hand throughout the latter half of the pointing movement (18). Subsequently, CPA mainly involves realignment of proprioceptive coordinates, whereas TPA relies more strongly on a strategic recalibration of visuomotor eye–hand coordinates. Although a few studies (13, 18, 29) have directly compared these 2 PA protocols, findings have been equivocal regarding which is more effective: CPA or TPA.

Despite previous studies (18, 26, 27) reporting factors that influence PA effects on USN, no meta-analyses focusing on these factors in combination have been reported. As previously mentioned by Szekely et al. (23), results from subgroup analyses must be interpreted carefully due to small sample sizes increasing the possibility of false positives. However, such analyses could help to optimize PA interventions and increase their clinical applicability, supporting a comprehensive, systematic, and evidence-based approach to addressing USN within stroke rehabilitation programmes. Furthermore, this may lead to patient benefits such as earlier improvements in USN symptoms, better functional outcomes, and further improvements in ADL.

To the best of our knowledge, this is the first meta-analysis to elucidate PA effects focusing on the prism angle, disease phase, and different PA protocols according to the visible range of own-hand movements. The purpose of this meta-analysis study is twofold: (*i*) to assess the short-term effects of PA upon clinical and functional outcomes of rehabilitation for USN after right hemispheric stroke, across studies with a more transparent and rigorous design, and (*ii*) to elucidate the contribution made by factors relating to the clinical and practical delivery of PA to short-term effects of PA.

## METHODS

This study was conducted in accordance with the Preferred Reporting Items for Systematic Reviews and Meta-Analyses (PRISMA) guidelines (30). The protocol for this systematic review and meta-analysis was prospectively registered with PROSPERO (CRD42024512755).

### Data sources and search strategy

The following online databases were used to search for randomized controlled trials, published in English up to 29 April 2024: PubMed, Cochrane Library, Web of Science, and CINAHL. In addition, some related studies included in previous systematic reviews (21–23) were manually retrieved and assessed for eligibility. The search was completed by 2 independent reviewers, and no disparity in results was identified.

The following combinations of the keywords were queried: “stroke” OR “cerebrovascular accident” OR “cva” OR “brain vascular accident” OR “apoplexy” OR “brain ischemia” OR “brain attack” OR “intracranial hemorrhage” OR “brain infarct” OR “cerebral accident” OR “brain damage” OR “brain lesion” AND “neglect” OR “visuospatial neglect” OR “visual neglect” OR “unilateral neglect” OR “hemisphere neglect” OR “sensory neglect” OR “unilateral spatial neglect” OR “spatial neglect” OR “visual inattention” OR “visuospatial neglect” AND “prism adaptation” OR “prism adaptive therapy” OR “prism glasses”. Citations detected by this search strategy were stored in Endnote X9 (Thomson Reuters, New York, USA).

### Study screening

Screening followed 2 steps:

- *Title/abstract screening*: 2 independent reviewers independently double-screened the titles and abstracts of the studies after eliminating duplicates. Inconsistencies in screening between reviewers were discussed and resolved.
- *Full text screening*: the reviewers independently screened the full text of the remaining studies using specific eligibility criteria. Discrepancies were resolved by a third reviewer where consensus could not be reached through discussion between reviewers.

### Eligibility criteria

*Patients:* Patients with USN after stroke who met the following criteria were included:

1. right hemispheric stroke patients (bilateral hemispheric stroke patients were excluded);
2. age 18 years old or above;
3. experience of a first stroke.

The exclusion criteria were:

1. a history of neurological or psychiatric disorders;
2. severe cognitive impairment.

### Intervention and comparator

Conservative rehabilitation treatment, such as physical and occupational therapy + PA, was included as an intervention group. Conservative rehabilitation treatment + sham, wearing neutral glasses and completing the same tasks, was included as a comparator (or control group). Studies that adopted passive control or conservative rehabilitative treatment only were excluded. PA interventions using any type of prism (Fresnel, wedge, and other) were included in this study. Lastly, only studies delivering 3 or more prism adaptation treatment sessions were included, as previous research indicates shorter treatment durations are not associated with clinically meaningful effects of PA (31, 32).

### Outcomes

The primary outcome was change in USN symptoms as measured by the conventional subtests of the Behavioral Inattention Test (BIT-C) (33, 34) or target cancellation tasks. The secondary outcome was change in Catherine Bergego Scale (CBS) (35, 36), an observational assessment often used to assess presence and degree of USN during ADLs.

### Data extraction

Two reviewers independently extracted the following data:

- *Study characteristics*: authors’ names and year of publication.
- *Patients*: sample size, age, gender, diagnoses, disease phase (acute, subacute, or chronic phases), severity of USN, inclusion/exclusion criteria.
- *Intervention and comparator*: prism angle, PA protocol, duration and dosage, blinding when performing pointing movement, contents of comparator training.
- *Outcomes*: means and standard deviations of primary and secondary outcome measures pre- and post-intervention, presence or absence of adverse events. If data were presented only as a graph, data were extracted using WebPlotDigitizer (https://automeris.io/WebPlotDigitizer).
- Others: statistical analysis, information related to the risk of bias, main conclusions.

Discrepancies between the two reviewers were resolved by discussion between the two reviewers until consensus was reached, or through intervention of a third reviewer.

### Risk of bias assessment

Two reviewers independently assessed the included studies using the Cochrane risk-of-bias tool (Version 2) (37). This tool is used to judge whether the selection, performance, detection, attrition, reporting, and other biases are “low risk”, “some concerns”, or “high risk”. Disagreements in the risk of bias assessment were resolved by discussion between the 2 reviewers until consensus was reached.

### Statistical analysis

A random-effect model was applied, to adequately account for additional uncertainty associated with heterogeneity between studies, using RevMan 7.2 (Cochrane Collaboration, Oxford, UK) (38) statistical software. Treatment effects were reported as standardized mean difference (SMD) with 95% confidence intervals (CIs). Statistical significance was reached when the 95% CI for the pooled SMD did not contain zero. Analysis was undertaken for primary (change in USN symptoms) and secondary (change in CBS) outcome measures to determine short-term effects of PA. In addition, subgroup analysis on each of prism angle, disease phase, and PA protocol (i.e., CPA, TPA) was undertaken to determine the extent to which each factor impacts primary and secondary analysis outcomes. Statistical heterogeneity in each analysis was assessed using the *I*^2^ statistic (39). Considering current recommendations regarding testing for funnel plot asymmetry (40), publication bias was only assessed using funnel plot asymmetry testing where more than 10 studies were available.

### Certainty of evidence for each meta-analysis

Two independent reviewers evaluated the certainty of evidence, using the Grading of Recommendations Assessment, Development and Evaluation (GRADE) methodology (41), for whole and subgroup analyses. The certainty of evidence was downgraded from high to moderate, low, and very low by rating each domain according to the following criteria: most studies are not low risk of bias, statistical heterogeneity *I*^2^ statistics exceed 50%, 95% CI for SMD is wide and sample size for meta-analysis is less than 400, there are studies of indirect comparisons, funnel plot is asymmetric (42).

## RESULTS

### Basic characteristics of included studies

**Fig. 1:**
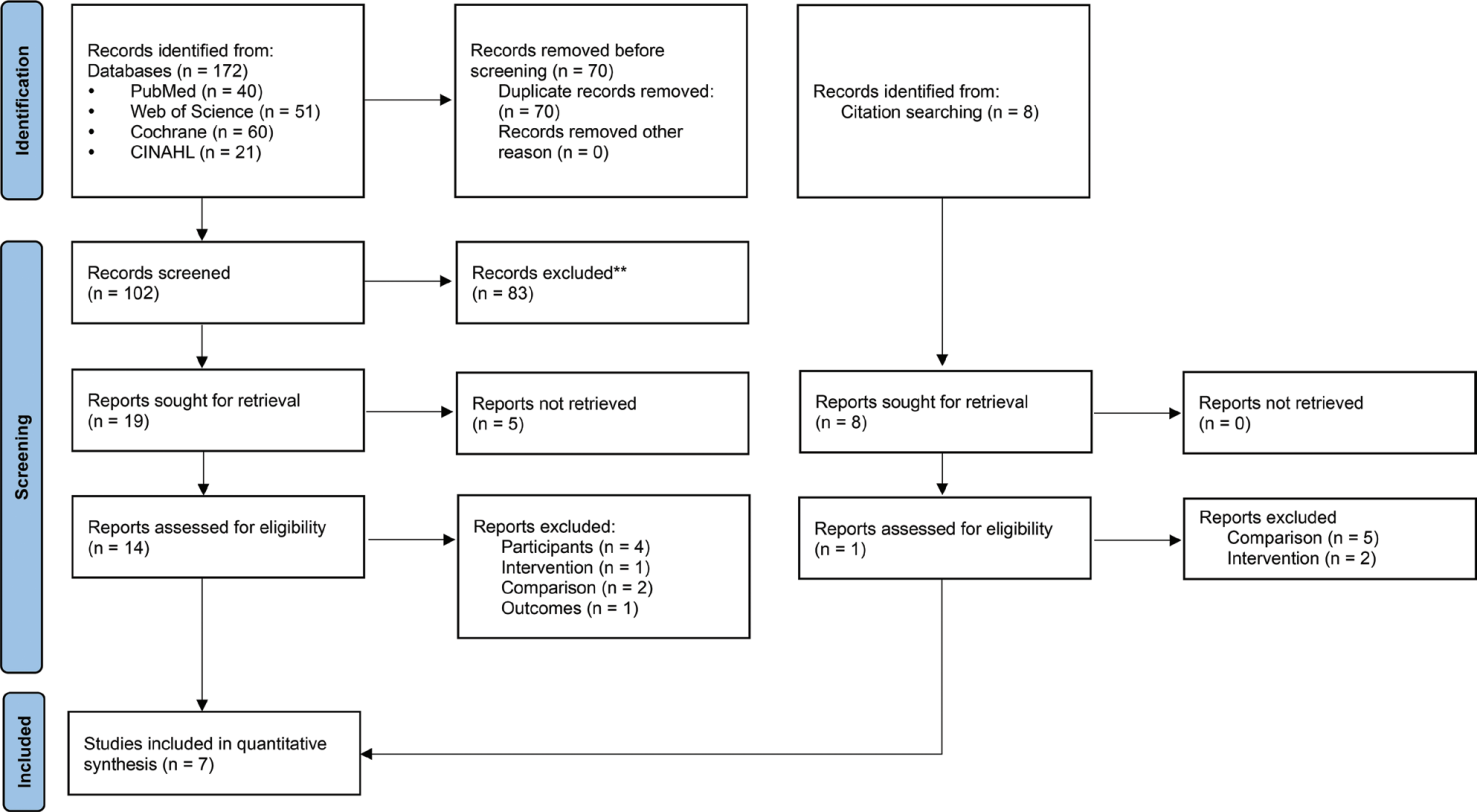
Preferred reporting items for systematic reviews and meta-analyses (PRISMA) flow diagram.

Fig. 1 shows the PRISMA flow diagram. Of the 180 retrieved studies, 7 RCTs (14, 18, 43–47) involving 227 participants satisfied the eligibility criteria. Table I shows the characteristics of the 7 included studies. The number of participants for primary and secondary outcomes, control treatment, group allocation, blinding, time after onset, length of full treatment, number of sessions, total time of sessions, the number of pointing movements during adaptation, strength of prism in degrees of visual angle, and type of exposure are reported.

**Table I:** Characteristics of all included studies (*n*=7)

Study	No. in neglect outcomes (PA/Control)	No. in CBS (PA/Control)	Group allocation	Blinding	Time after onset (days)	Total treatment duration (days)	No. of sessions	Total time of sessions (min)	No. of pointing movements	Prism strength (°)	PA procedure
Nys, 2008 (43)	10/6	N/A	Random	Blinding to patients	8.8 ± 5.3/acute	4	4	120	100 left and right	10	CPA
Serino, 2009 (44)	10/10	N/A	Pseudo-random	Blinding to patients	9.6 ± 18 months/chronic	14	10	300	90 left, right and center	10	TPA
Turton, 2010 (45)	16/18	16/18	Pseudo-random	Blinding to assessors	45 ± 23/subacute	14	10	N/A	30 left, right and center	6	TPA
Mizuno, 2011 (46)	13/18	15/18	Random	Double-blind	67.1 ± 18.4/subacute	14	20	400	90 to 3 targets	12	TPA
Ladavas, 2011 (18)	20/10	N/A	Pseudo-random	Double-blind	7.0 ± 7.8 months/ subacute and chronic	14	10	300	90 left, right and center	10	CPA, TPA
Mancuso, 2012 (47)	13/9	N/A	Random	Blinding to patients	180.2 ± 301.5 301.485/chronic	7	5	150	90 left, right and center	5	TPA
Umeonwuka, 2022(14)	37/37	37/37	Random	Double-blind	42(26–56)/subacute	16	12	360	90 to 3 targets	11.4	TPA

PA: prism adaptation; N/A: No data available; CPA: concurrent prism adaptation; TPA: terminal prism adaptation.

Regarding clinical and practical considerations for PA, 2 studies (44, 47) examined the effects of PA during the chronic phase of stroke rehabilitation, while the others investigated the acute or subacute phases. Two studies (45, 47) adopted a small prism angle (< 10° [≈ 17.5 Δ prism]), and the others used a large prism angle (≥ 10°). Two studies used CPA (18, 43), and the others used TPA.

The total time of PA treatment ranged from 120 to 400 min, and most studies applied a 2-week treatment duration.

For time after stroke onset, 2 studies (18, 44) are described by month. The following definitions for post-stroke stages were used: the first 2 weeks post-stroke are termed as the acute stage, 3–11 weeks post-stroke is termed the subacute stage, in which most changes occur; more than 12 weeks post-stroke is the chronic stage (48). The total time of sessions (min) is calculated from the number of sessions and time per session, where time per session is known.

### Risk of bias

**Fig. 2:**
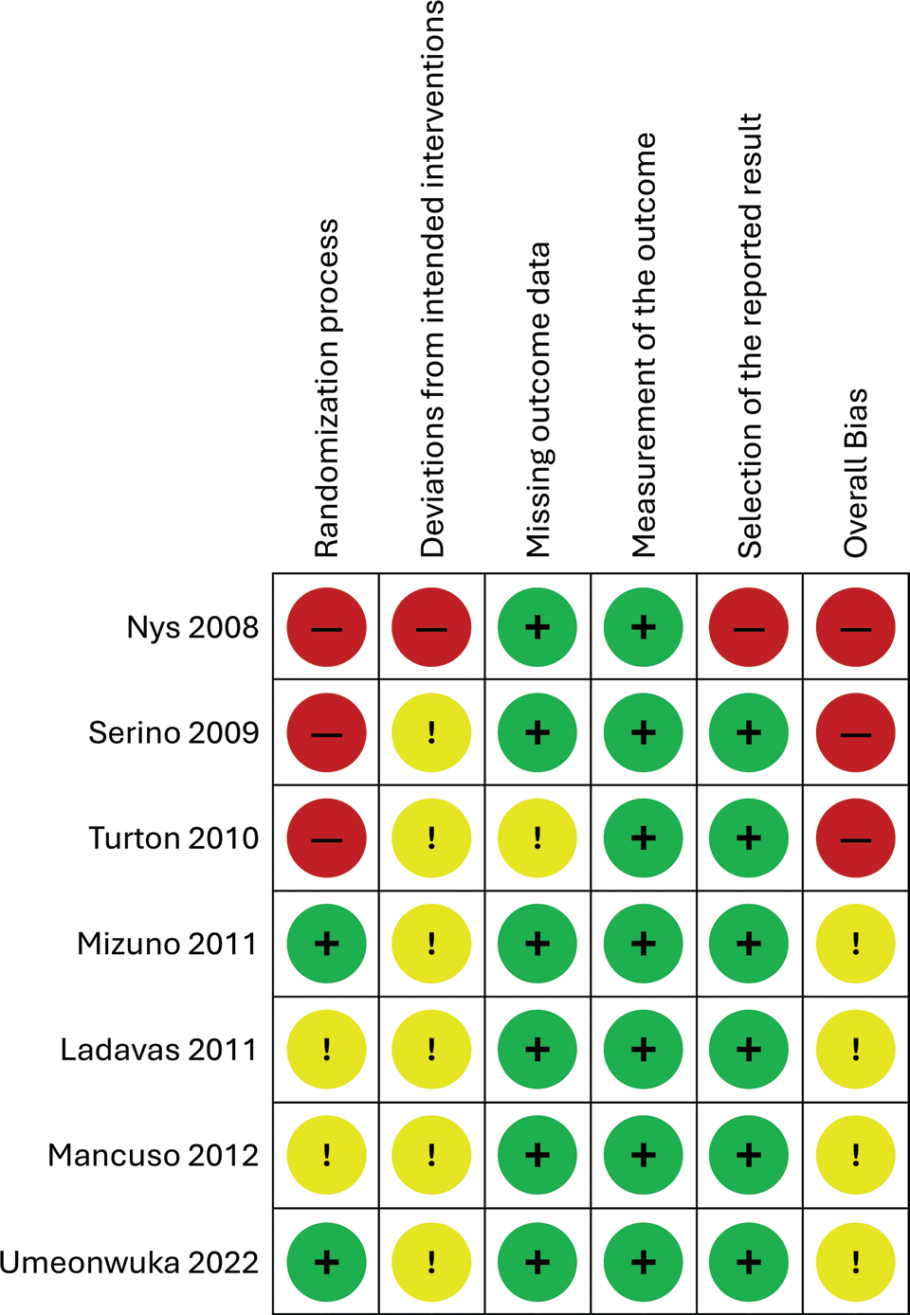
Risk of bias summary for all included studies. Symbols: +: low risk; !: some concerns; -: high risk.

The summary of the risk of bias assessment is shown in Fig. 2. Among the 7 studies, none were judged as having a low risk of bias. Four studies (14, 18, 46, 47) were judged to have some concerns, and the other 3 studies (43–45) were judged as having a high risk of bias. The latter studies were judged as high risk due to the lack of information concerning the randomization process. For deviation from the intended intervention and selection of the reported results, most of the studies were judged as having some concerns.

### Meta-analysis

**Fig. 3:**
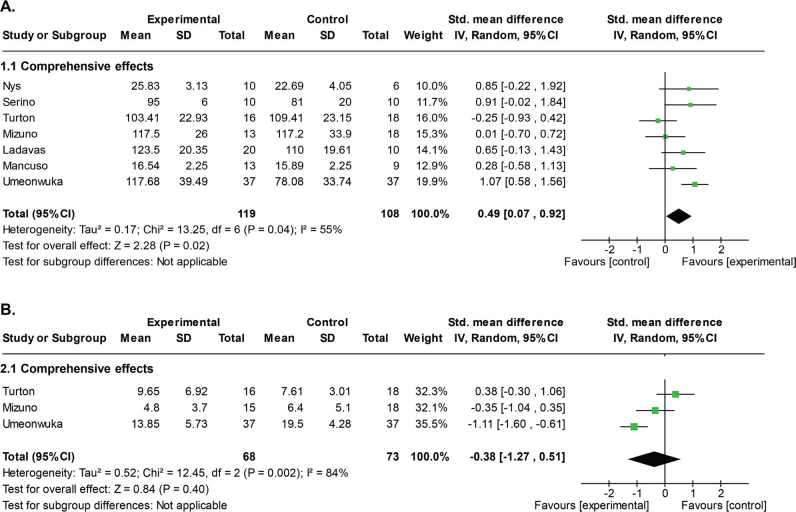
Forest plots of the random effect models on the short-term PA effects for the neglect outcomes and CBS. (A) Comprehensive effects for the neglect outcomes. Two studies (43, 47) used cancellation tasks and other studies used BIT-C. (B) Comprehensive effects for the CBS. These forest plots were generated by RevMan 7.2.

Fig. 3 shows the forest plots for the primary (USN change) (Fig. 3A) and secondary (CBS change) (Fig. 3B) outcome measures, respectively. Table II shows the certainty of the evidence and summary of findings for all included studies. All studies included the primary outcome measure. To measure these USN symptoms, 5 (14, 18, 44–46) employed the BIT-C, and 2 (43, 47) utilized a cancellation task. A random-effect model revealed a statistically significant short-term effect of PA (SMD: 0.49 [95% CI: 0.07 to 0.92], *p* = 0.02, *I*^2^ = 55%, very low certainty evidence) (Fig. 3A and Table II).

**Table II:** Certainty of the evidence and summary of findings

Meta-analysis	Risk of bias	Inconsistency	Indirectness	Imprecision	Publication bias	Certainty
Neglect outcomes						
Comprehensive effects	Serious	Serious	Not serious	Serious	NA	Very low
Subgroup analysis						
Disease phase acute subacute	Serious	Serious	Not serious	Serious	NA	Very low
Disease phase chronic	Serious	Not serious	Not serious	Serious	NA	Low
Terminal PA	Serious	Serious	Not serious	Serious	NA	Very low
Concurrent PA	Serious	Not serious	Not serious	Serious	NA	Low
Prism angle larger	Serious	Not serious	Not serious	Serious	NA	Low
Prism angle smaller	Serious	Not serious	Not serious	Serious	NA	Low
CBS						
Comprehensive effects	Serious	Serious	Not serious	Serious	NA	Very low
Subgroup analysis						
Disease phase acute subacute	Serious	Serious	Not serious	Serious	NA	Very low
Disease phase chronic	Serious	Serious	Not serious	Serious	NA	Very low
Prism angle larger	Serious	Serious	Not serious	Serious	NA	Very low

NA: not applicable; PA: prism adaptation; CBS: Catherine Bergego Scale.

Three studies (14, 45, 46) included in the meta-analysis collected the secondary outcome measure. There was no statistically significant difference between the 2 groups (SMD: –0.38 [95% CI: –1.27 to 0.51], *p* = 0.40, *I*^2^ = 84%, very low certainty evidence) (Fig. 3B and Table II). As fewer than 10 papers were included in these analyses, no funnel plot analysis was applied.

### Subgroup meta-analysis

**Fig. 4:**
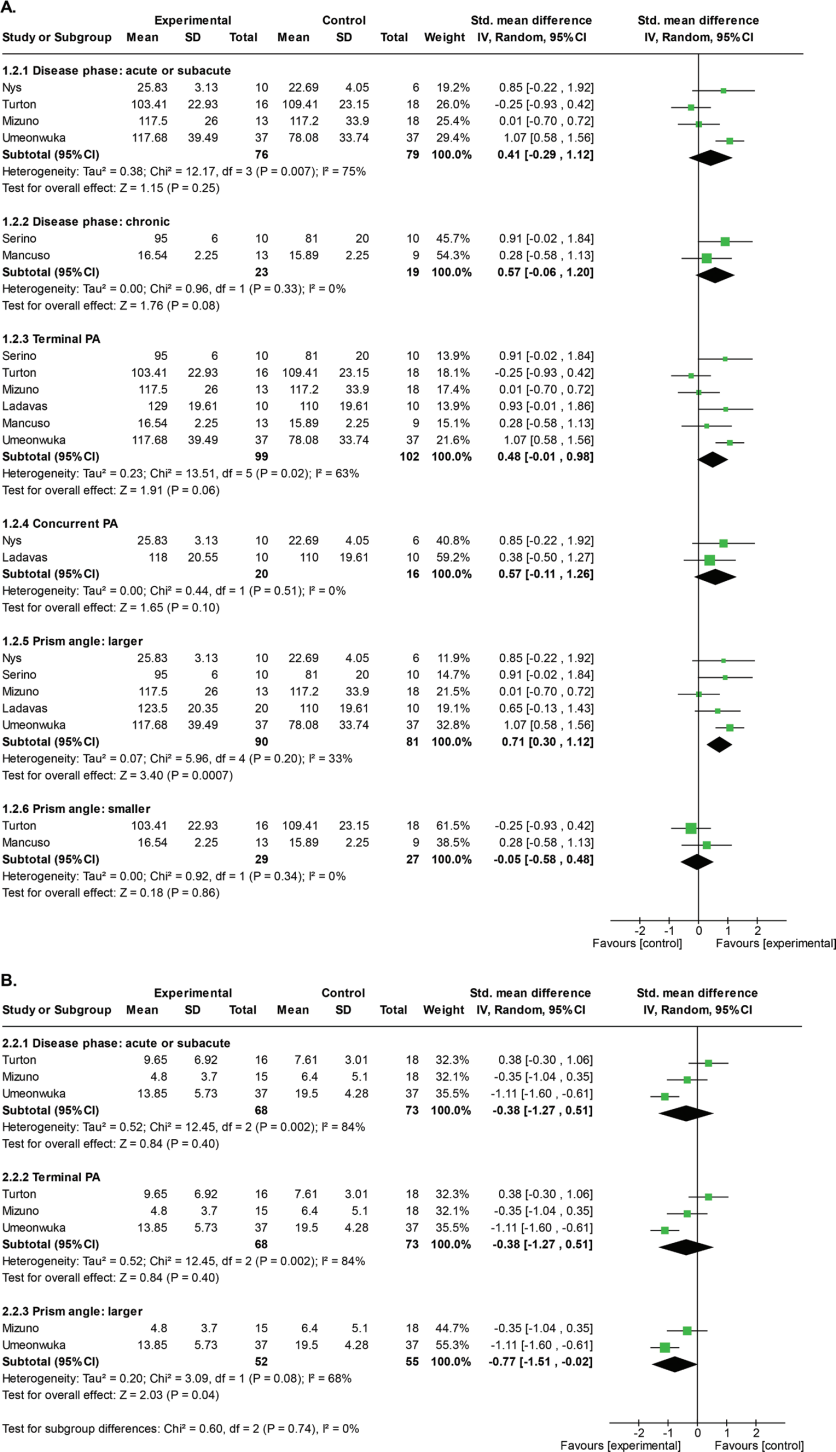
Forest plots of the random effect models on the short-term PA effects in the subgroup analysis of neglect outcomes and CBS. (A) Factor-specific effects of the neglect outcomes. (B) Factor-specific effects of the CBS. These forest plots were generated by RevMan 7.2.

Fig. 4 presents the forest plots illustrating the subgroup analyses for the primary and secondary outcomes.

In the disease phase, classification was conducted based on whether the condition was in the acute, subacute phase, or chronic phase, as previously defined. One study (18) was excluded because it recruited the participants across the subacute and chronic phases. This resulted in an acute/subacute phase condition with 4 studies (14, 43, 45, 46) and a chronic phase condition with 2 studies (44, 47). There were no statistically significant differences between the PA and control groups for either condition, but a trend towards statistical significance for larger effects in the PA group was observed in the chronic phase (acute/subacute phases: SMD: 0.41 [95% CI: –0.29 to 1.12], *p* = 0.25, *I*^2^ = 75%, very low certainty evidence; chronic phase: SMD: 0.57 [95% CI: –0.06 to 1.20], ***p*** = 0.08, *I*^2^ = 0%, low certainty evidence) (**Fig. 4**A and Table II).

For PA protocol (i.e., hand visibility during pointing), classification was performed based on whether TPA or CPA was employed. Five studies (14, 18, 44–47) used TPA, and 2 studies (18, 43) used CPA. There were no statistically significant differences between the PA and control groups for either condition (terminal PA: SMD: 0.48 [95% CI: –0.01 to 0.98], *p* = 0.06, *I*^2^ = 63%, very low certainty evidence; concurrent PA: SMD: 0.57 [95% CI: –0.11 to 1.26], *p* = 0.10, *I*^2^ = 0%, low certainty evidence) (Fig. 4A and Table II).

For prism angle, studies were classified into 2 conditions, based on the criterion of whether the prism angle was greater than or less than 10°. A larger angle (≥ 10°) was used in 5 studies (14, 18, 43, 44, 46) and a smaller angle (< 10°) in 2 studies (45, 47). The larger angle group showed a statistically significant short-term PA effect on the primary outcome measure (SMD: 0.71 [95% CI: 0.30 to 1.12], *p* = 0.0007, *I*^2^ = 33%, very low certainty evidence) (Fig. 4A and Table II) whereas there were no statistically significant effects for the smaller angle condition (SMD: –0.05 [95% CI: –0.58 to 0.48], p = 0.86, *I*^2^ = 0%, low certainty evidence) (Fig. 4A and Table II).

For the secondary outcome measure, only subgroup analysis of prism angle was conducted, as all 3 studies collecting this outcome measure were classified into the same condition for stage post-stroke (acute/subacute) and PA protocol (TPA).

For prism angle, classification was performed in the same way as for the primary outcome analysis. This resulted in 2 larger angle studies (14, 46) and 1 smaller angle study (45), thus meta-analysis was performed only for the former group. Statistically significant short-term PA effects were identified for the secondary outcome measure for this group (SMD: –0.77 [95% CI: –1.51 to –0.02], *p* = 0.04, *I*^2^ = 68%, very low certainty evidence) (Fig. 4B and Table II).

## DISCUSSION

This study was a pre-registered systematic review and meta-analysis to consolidate the literature on use of PA within rehabilitation for USN, following right hemispheric stroke. Across 7 studies that satisfied eligibility criteria, we examined the short-term effects of PA upon clinical outcomes (USN symptoms, measured using conventional neuropsychological assessments), and functional outcomes (measured using CBS), and analysed the impacts of different PA effect factors such as prism angle and PA protocol through subgroup analysis. This is the first report of subgroup analysis in this context (USN following right hemispheric stroke), focusing on 3 important factors (prism angle, PA procedure, and stage of disease). Three previous meta-analyses have examined the effects of PA on USN (21–23). Compared with those studies, the inclusion criteria of the present study were more strictly controlled as follows: focusing on patients with an initial onset and right hemispheric stroke and assigning the control group as active-sham control (performing the pointing task with neutral glasses). The inclusion of a more recent study by Umeonwuka and colleagues (2023) (14) makes this an up-to-date meta-analysis. Another strength is to use the GRADE methodology to assess evidence certainty, which previous studies (21–23) did not utilize.

Regarding concurrence of findings with other meta-analyses, the lack of short-term PA effects observed for the CBS outcome measure aligns with findings of 5 other studies (19–23). However, the improvement noted in the BIT was consistent with 2 previous studies (19, 21), which had similar eligibility criteria to our own. However, control group criteria in 1 of these studies (21) were less stringent by comparison, as it included passive controls, and the other study (19) was published a decade ago, thus omitting recent papers. Furthermore, the 2 systematic reviews (24, 25) that did not conduct a meta-analysis reported positive effects of PA on USN symptoms. However, each of these reviews focused on different outcome measures – visual search performance (24) and functional outcomes (25) – making it challenging to draw consistent and reliable conclusions regarding the overall effectiveness of PA. As we conducted a meta-analysis designed to rigorously address these issues, findings from our study help to address these inconsistencies with greater reliability.

This study revealed a significant short-term improvement following PA in the primary outcome measure (USN symptoms), but not in the secondary outcome measure (CBS) in this comprehensive key analysis. Most studies on PA focus on short-term effects, with few reports verifying long-term effects. Among these limited studies, long-term effects on reducing USN symptoms were observed only in the PA intervention group, maintained for 5 weeks post-treatment (12). Notably, these long-term effects were shown only in patients who exhibited short-term effects immediately after PA. These findings suggest that PA is effective for longer-term improvements in USN symptoms, with short-term effects being a prerequisite for long-term benefits.

Moreover, subgroup analysis demonstrated the statistically significant effects of PA for larger prism angles (prism angle ≥ 10°) for primary and secondary outcome measures, whereas there were no such effects upon the primary outcome measure for smaller prism angles. Meanwhile, null effects were found for impact of intervention timing, in terms of stage post-stroke, and PA protocol. Detection of statistically significant effects of PA upon USN symptoms but not CBS in the main analysis may be explained as follows:

1. CBS has inter-rater variability due to therapist subjectivity and USN oversight (49).
2. Heterogeneity of the CBS indicating greater variability in ratings across studies, as demonstrated in the present analysis.
3. PA effects may not extend to significant improvement of everyday life behaviour. Some previous studies support this possibility (21, 27, 50), but given the known impact of USN upon rate of improvement in functional independence measures, it may be that CBS is simply not a sensitive outcome measure for short-term effects of PA. The results of the present study suggest that use of PA within rehabilitation for USN has limited short-term effects upon functional outcomes as measured with a commonly used instrument (CBS). This underscores the importance of using other assessments to mitigate potential issues with using the CBS in isolation, and larger sample sizes with longer follow-up may be needed to better understand the nature of PA effects upon functional outcomes of rehabilitation for USN. Using the Kessler Foundation Neglect Assessment Process (KF-NAP) (51) may be reasonable to minimize the inter-rater variability and heterogeneity of the CBS across studies because KF-NAP standardizes administration and strengthens the reliability of CBS, potentially contributing to this solution.

Regarding the subgroup analyses, statistically significant PA effects were demonstrated for the primary and secondary outcome measures when larger prism angles were used (≥ 10°). No prior meta-analyses or systematic reviews (19–25) have reported significant effects on both clinical and functional outcomes; this study is the first to demonstrate significant effects on both clinical and functional outcomes. Some previous studies supported this effect: prism angles greater than 10° were more likely to produce aftereffects in USN patients (26), and were required to produce PA effects in the cognitively normal subjects (52). The degree of visual error induced by PA has been suggested as an important factor in magnitude of the strategic realignment process (correction of errors in motor output), and the resulting shift of the oculomotor system that can lead to improvement in USN symptoms (18). Therefore, prism angles exceeding 10° may be a key contributor to the effectiveness of PA as USN treatment, the benefits of which could potentially permeate into the level of everyday life behaviour. Future trials should consider employing PA with a prism angle of 10° or more. By contrast, there were no statistically significant differences in outcome measures by PA protocol (CPA or TPA), suggesting that choice of PA protocol does not have a factor-specific effect on the treatment response. Previous studies (13, 18, 29) directly comparing TPA and CPA groups were equivocal on which PA protocol was superior. A trend towards statistical significance was observed in our study for TPA, and only 2 studies on CPA were included, which may have been insufficient to observe the effects adequately. Therefore, the impact of choice of protocol (TPA or CPA) on the effectiveness of PA remains equivocal. Further studies are required to determine which is more effective. As with the PA protocol, no statistically significant differences in outcome measures were observed in the subgroup analysis of intervention timing by stage post-stroke. Evaluating outcomes of patients in the chronic phase has the advantage of not being affected by spontaneous recovery, and the effectiveness of PA in this phase was shown in some previous studies (12, 53, 54). However, only 2 studies classified into chronic phase met the strict eligibility criteria in this study. Consequently, it is difficult to draw conclusions concerning PA effects in the chronic phase within this study, and there is a need for further, rigorous intervention studies for PA in this phase. In the acute phase, PA effects might be difficult to detect due to spontaneous recovery and large symptom variability (55–57), and manifestation of cognitive and physical symptoms immediately after onset (58). This may explain the findings of the present subgroup analysis.

Several limitations should be considered. First, as only 7 papers were eligible for inclusion, some subgroups lacked sufficient data for analysis. Second, most of the main and subgroup analyses were deemed to have low or very low certainty of evidence. Third, some studies provided only graphical representations of results, and were calculated using WebPlotDigitizer, potentially affecting results. Fourth, in some subgroup analyses, the number of studies included was as few as 2. It should be noted that these results may change after more studies are conducted. Finally, only articles published in English were included, increasing the risk of publication bias.

In conclusion, the present study comprehensively examined short-term effects of PA within rehabilitation of USN. This meta-analysis employed stricter inclusion criteria, focusing on well-controlled studies and clinically meaningful session counts to identify gaps and make suggestions regarding future studies to optimize the clinical approach to PA. This analysis demonstrated significant short-term effects of PA on the primary outcome measure of USN symptoms, despite overall low certainty of evidence. Specifically, PA using prism angles greater than 10° yielded statistically significant improvements in both USN symptoms and activities of daily living. These findings support the use of PA with prism angles exceeding 10° within rehabilitation for USN after right hemispheric stroke. In addition, these observations may explain contradictory results obtained from previous studies. Future RCTs should consider employing PA with a larger prism angle, greater sample sizes, and longer follow-up periods with a focus on the chronic phase of stroke recovery, to develop the evidence base for effectiveness and optimization of PA, advancing beyond previous findings.
